# Table of the Pulses in Diseases of the Heart

**Published:** 1849-12

**Authors:** 


*Table of the Pulses in Diseases of the Heart.*—

1. *Simple hypertrophy of the left ventricle:* Strong and prolonged.

2. *Hypertrophy with dilatation:* Strong, prolonged, and large.

The above pulses, when accelerated, become hard.—They may become weak and small, by any cause which enfeebles the heart's action.

3. *Hypertrophy with contraction:* Tense, but small.

4. *Dilatation, with hypertrophy:* The former predominating. Large and prolonged, but soft, often accelerated, "bounding."

5. *Dilatation, with attenuation:* Large and weak; small in the latter stages.

6. *Softening:* Small, weak, more or less irregular, unequal, and intermittent.

7. *Free regurgitation through the aortic valves:* Eminently "jerking," the arteries unfilled.

8. *Contraction of the aortic valves:* Strength little impaired, unless the contraction be considerable. Regularity generally unaffected.

9. *Great contraction of, or free regurgitation through the mitral valves:* Small, weak, irregular, intermittent, and unequal.

10. *Polypus formed before death: Endocarditis with polypus*, and pericarditis with effusion—all cause suddenly a small, weak, irregular, and intermittent pulse.—*Hope on Diseases of the Heart*, 4th edition.

